# Emerging viral diseases: an Australian perspective.

**DOI:** 10.3201/eid0501.990101

**Published:** 1999

**Authors:** J S Mackenzie

**Affiliations:** University of Queensland, Brisbane, Australia. jmac@biosci.uq.edu.au


### Emerging Viral Diseases: An Australian Perspective

**John S. Mackenzie**
The University of Queensland, Brisbane, Australia

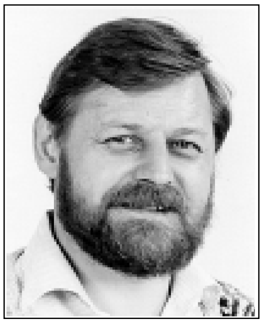

Dr. Mackenzie is professor and head of the Department of Microbiology and Parasitology, University of Queensland, Brisbane, Australia. His research interests include the epidemiology, ecology, and molecular biology of mosquito-borne and emerging zoonotic viruses.

With a few exceptions, emerging diseases in Australia are similar to those in other industrialized countries (1-8). Most exceptions are either vector-borne or zoonotic viral diseases, the major focus of this update. The continuing emergence of antibiotic resistance is a worldwide problem. In Australia, antibiotic resistance is being reported from a growing number of organisms (9-15), often necessitating new case management practices and guidelines (16,17). Also, like other countries, Australia has had a number of foodborne (18-22) and waterborne (23-25) epidemics in the past few years; the major difference is that the higher incidence of enterohemorrhagic *Escherichia coli* linked to outbreaks of hemolytic uremic syndrome is associated with serotype O111:H- rather than serotype O157:H-, which is more common in other countries. Major waterborne epidemics or contamination of reservoirs due to *Cryptosporidium parvum* have occurred over the past 3 years in the Eastern States of Australia (23-25), with the largest and most recent being a problem of contamination (in association with *Giardia lamblia*) in the Sydney water supply between July and September, 1998. However, despite this contamination, no increases in the number of cases of diarrheal disease were reported, perhaps because the inhabitants of Sydney were advised to boil the water before drinking it (25).

The distribution and incidence of most of the recently described viral diseases (e.g., human herpesviruses 6–8 and hepatitis C and E viruses) in Australia are similar to those reported in other industrialized nations (3). Recent data for hepatitis G in selected Australian populations also support this contention (26).

### Vector-Borne Viral Diseases

Australia has more than 70 arboviruses, but relatively few cause human disease (27,28). The most common arbovirus causing human disease is Ross River virus, an alphavirus, which causes an epidemic polyarthritis. Although Ross River virus incidence has increased over the past decade, the virus is not emerging; its increased incidence is probably due to increased awareness and recognition by general practitioners, improved diagnostic reagents, and increasing encroachment of human habitation into or near wetlands and other areas conducive to mosquito breeding. The only indigenous virus that can be called "emerging" is Barmah Forest virus, also an alphavirus and also the cause of an epidemic polyarthritis-like disease. Associated with human disease only since 1988 and increasing in incidence as diagnostic reagents have become available and clinicians have become aware of it, the virus has spread into new geographic areas, such as Western Australia (29,30). The two mosquito-borne diseases of particular concern, however, are "imports"—Japanese encephalitis (JE) and dengue viruses.

### Japanese Encephalitis Virus

The first outbreak of JE in the Australian region occurred in Torres Strait, northern Australia, in 1995 (31). Three cases (two of which were fatal) were reported from Badu Island in central Torres Strait, 2,000 km from the nearest

Address for correspondence: John S. Mackenzie, Department of Microbiology, The University of Queensland, Brisbane, Qld 4072, Australia; fax: 61-7-3365-4620; e-mail: jmac@biosci.uq.edu.au.

focus of JE virus activity in Bali. Seroepidemiologic studies showed that the virus was relatively widespread in the central and northern islands with subclinical human cases on four islands and seropositive pigs on nine islands. Ten virus isolates were obtained during the outbreak: two from subclinical human infections (31) and eight from *Culex annulirostris* mosquitoes collected on Badu Island (32). Sequencing studies showed that these isolates (most closely related to a 1970 isolate from Kuala Lumpur and a 1981 isolate from Bali) (33) were almost identical, suggesting that the outbreak originated from a single source. These studies also showed that all isolates had the same 11 nucleotide deletion in the 3' untranslated region immediately downstream from the stop codon of the open reading frame (34), which provided a signature for comparing any future isolates. After the Badu Island outbreak, inactivated vaccine was offered to all the inhabitants of the northern and central Torres Strait islands (35). During 1996 and 1997, JE virus activity was detected through seroconversions in sentinel pigs on Saibai Island, which is in the north and only about 4 km from the Papua New Guinea coast (36) (J. Lee, D. Phillips, J. Hanna, unpub. results).

Seroepidemiologic studies found that virus activity has been widespread in Western Province, Papua New Guinea, since at least 1989, with seropositivity rates of 21% at that time among the Daru-speaking people. Results also showed that seropositivity rates were increasing in the Upper Fly River area and that the virus was spreading geographically (37). Indeed, recent results indicate that JE may have spread to Vanimo on the northern coast by April 1998 and to parts of Milne Bay Province in eastern Papua New Guinea (J. Lee, J. Wangi, P. Siba, G. Tau, unpub. results). The first four clinical cases of JE in Papua New Guinea were observed in 1997 and 1998, with two deaths. All cases were from the Kiunga area of Western Province (J. Oakley, S. Flew, C.A. Johansen, D. Phillips, R.A. Hall, J.S. Mackenzie, unpub. results). Anecdotal evidence suggests that the cases may have resulted in part from the large mosquito numbers associated with the severe drought in 1997. The first JE virus strain isolated in Papua New Guinea was obtained from *Cx. annulirostris* mosquitoes collected at Lake Murray in Western Province in 1997. Sequence studies have shown that this isolate was almost identical to the 1995 Torres Strait isolates, including the acquisition of the 11 nucleotide deletion (C.A. Johansen, R. Paru, S.A. Ritchie, A. Van den Hurk, M. Bockarie, J.S. Mackenzie, unpub. results).

A second outbreak of JE occurred in Torres Strait in March 1998, with one human case from Badu Island and sentinel pigs seroconverting on a number of islands. Shortly afterwards, the first human case on mainland Australia was reported in a fisherman who acquired the infection near the mouth of the Mitchell River in southwest Cape York (38). Extensive seroepidemiologic investigations found no further human infections in communities on Cape York, but domestic pigs had seroconverted both near Mitchell River and near Bamega at the northerly tip of Cape York. Two virus isolations were obtained from pig sera collected near Bamega, and one isolate was obtained from a sentinel pig on Mabuiag Island in Torres Strait (J. Hanna, S. Hills, D. Phillips, J. Lee, unpub. results). Mosquitoes were collected at a number of sites on Cape York as well as on Badu Island. No viruses were isolated from the Cape York mosquitoes, but approximately 44 isolates were obtained from Badu Island—43 from *Cx. annulirostris* mosquitoes and one from *Aedes vigilax* mosquitoes (S.A. Ritchie, A. Van Den Hurk, C.A. Johansen, D. Phillips, A. Pyke, J.S. Mackenzie, unpub. results). Nucleotide sequencing studies have shown that the mosquito and pig isolates from Mabuiag and Cape York were closely related to each other, as well as to the 1997 Papua New Guinea Lake Murray and the 1995 Badu Island isolates, including all isolates sharing the 11 base “signature” deletion, which indicated a single virus source for the virus activity in Northern Australia and Papua New Guinea. The focus of activity is probably in Papua New Guinea (C.A. Johansen, A. Drew, D.A. Phillips, A. Pyke, J.S. Mackenzie, unpub. results).

JE virus activity in northern Australia began in 1998. Sentinel animal sites are being established to investigate whether the virus has become enzootic in the wildlife. Australia has both the mosquito vectors (*Cx. annulirostris*) and vertebrate hosts (ardeid birds and pigs) for the virus to become established. In addition, large areas of wetland habitats in Cape York would be conducive to virus enzootic cycles and would increase the potential for the virus to move south to more populous areas of Australia (39).

### Dengue Viruses

Despite a 120-year history, dengue does not appear to be endemic in Australia. Several epidemics over the past decade have been initiated from virus introduced by viremic travelers (27,28,40). Imported cases of dengue in travelers are regularly diagnosed throughout Australia, with 30 to 60 cases reported annually, and growing in number. In most parts of Australia where the local mosquitoes are unable to transmit dengue viruses, these cases pose no risk, but in areas of north Queensland where *Ae. aegypti* is common and travel between Australia and countries in the Asian-Pacific area is frequent, local transmission and epidemic activity are major risks. The potential for local transmission of dengue viruses is confined to an area of Queensland corresponding to the geographic range of *Ae. aegypti*, extending from the islands of Torres Strait in the north, to Mount Isa and Boulia in the west, possibly to Roma in the south, and to Gladstone on the east coast (41). Despite this relatively broad geographic range, epidemic activity over the past 2 decades has been restricted from Torres Strait south to Cairns, Townsville, and Charters Towers. The major epidemics over the past 5 years have included a large outbreak of dengue type 2 in 1992 to 1993, principally in Townsville and Charters Towers with more than 2,000 cases, and with the first case of dengue hemorrhagic fever this century (42,43); an outbreak of dengue type 2 in 1996 to 1997 on a number of Torres Strait islands and Cairns, with more than 200 serologically confirmed cases (44,45); and an outbreak of dengue type 3 in 1997 to 1998 largely restricted to Cairns with 239 confirmed cases (46; S. Ritchie, S. Hills, pers. comm.) and also a few cases of dengue type 2. This latter outbreak also included a case of dengue hemorrhagic fever and the first case of dengue encephalopathy in Australia (J. Hanna, unpub. obs.). Nucleotide sequencing of dengue 2 isolates from Australia and a comparison with isolates from elsewhere in the Asian-Pacific region indicated that the 1992-93 isolates were most closely related to an Indonesian virus, whereas the 1996-97 isolates were most closely related to viruses originally isolated in Burkina Faso. This latter finding is of interest because a large outbreak of dengue type 2 occurred on a number of Pacific Islands before and during the Australian outbreak, but the South Pacific viruses were quite distinct from the Australian viruses (45).

After the 1992-93 outbreak, a Dengue Fever Management Plan was developed to reduce the potential for epidemic activity from imported cases. The plan has been extremely successful, and a number of imported cases have been recognized early and were contained before they could cause an epidemic (47,48). However, importation, either by continual movement of people between Papua New Guinea and Torres Strait or by movement of people for work, education, or recreation between Papua New Guinea and north Queensland, will always be a major route of entry of the virus.

Vector importations occur frequently, with a number of reports for both *Ae. aegypti* and *Ae. albopictus* (27), including two recent importations of *Ae. albopictus* into Townsville in 1997 (49) and Cairns in 1998 (S. Ritchie, pers. comm.).

### Novel Zoonotic Viral Diseases

In the past 4 years, three newly described zoonotic viral diseases have been reported from Australia; two of these diseases are caused by the paramyxoviruses Menangle and Hendra (formerly equine morbillivirus), and the third is caused by Australian bat lyssavirus.

### Menangle Virus

An apparently new virus in the family *Paramyxoviridae* was isolated from stillborn piglets with deformities at a large commercial piggery in New South Wales (51). The farrowing rate in the piggery decreased from an expected 82% to 60%; the number of live piglets declined in 27% of the litters born; the proportion of mummified and stillborn piglets, some with deformities, increased; and occasional abortions occurred. Virus was isolated from lung, brain, and heart tissues of infected piglets, and shown to be morphologically similar to viruses in the family *Paramyxoviridae*. No disease was seen in postnatal animals of any age, but a high proportion of sera (>90%) from animals of all ages contained high titers of neutralizing antibodies against the virus. Tests performed at the Australian Animal Health Laboratory confirmed that the virus, named Menangle virus, was unrelated to other known paramyxoviruses, including viruses known to infect pigs (51).

Serum from two workers—one at the affected piggery and one at an associated piggery that had received weaned pigs from the original piggery—had high titer, convalescent-phase neutralizing antibodies to the new virus. Both workers had an influenzalike illness with rash during the pig outbreak, but extensive serologic testing showed no evidence of any alternative cause; therefore, the illness is believed to have been caused by the new virus (52).

A large breeding colony of gray-headed and little red fruit bats roosted within 200 m of the affected piggery. In a preliminary study, 42 of 125 serum samples collected from fruit bats in New South Wales and Queensland had neutralizing antibodies to the new virus. In addition, antibodies were found in sera collected in 1996, before the outbreak, and from a colony of fruit bats 33 km from the piggery (51). Therefore, the fruit bats are believed to be the primary source of virus causing the outbreak. Sera collected from wild and domestic animals near the affected piggery were seronegative.

The geographic range, normal host species, and genetic relationship of this new virus to other paramyxoviruses remain unknown. Nevertheless, Menangle appears to cause fatal disease and malformations in prenatal pigs and may be associated with influenzalike illness in humans.

### Hendra Virus

Hendra virus was first recognized in 1994 after an explosive outbreak of severe, fatal respiratory disease affecting race horses and humans. Twenty race horses in the Brisbane suburb of Hendra were infected; 13 died. The trainer and stable hand were also infected, and the trainer died (53-55). A second incident occurred in Mackay, a coastal town approximately 1,000 km north of Brisbane. Two horses and a farmer died, the latter from severe meningoencephalitis (56-58). The death of the horses and the initial infection of the farmer occurred in 1994 and preceded the Brisbane outbreak; the virus is believed to have then entered a latent phase for 1 year before reactivating to cause fatal encephalitis. No connection was found between the Brisbane and Mackay incidents (56). Experimental studies have shown that in horses and cats, after subcutaneous, intranasal, and oral administration, the virus causes fatal pneumonia (59). In guinea pigs, subcutaneous administration is also fatal, but the infection is more generalized. Black fruit bats (*Pteropus alecto*) infected by subcutaneous, intranasal, or oral routes contract a subclinical infection and generate an antibody response (M. Williamson, unpub. results). Endothelial cell tropism and formation of syncytia in blood vessels are common pathologic findings in both overt and subclinical infections (B.T. Eaton, M. Williamson, unpub. obs.).

Extensive seroepidemiologic studies found no evidence of Hendra virus among horses, other farm animals, or more than 40 species of wildlife in Queensland (56,60; P.L. Young, K. Halpin, H. Field, unpub. results). However, working on the hypothesis that if outbreaks at two distant sites were connected, the most likely wildlife source would be either birds or fruit bats, P.L. Young and colleagues subsequently showed that fruit bats (flying foxes), members of *Megachiroptera*, were the natural hosts on serologic grounds and by virus isolation, with widespread evidence of infection in four species of fruit bat: the black (*Pteropus alecto*), grey-headed (*P. poliocephalus*), little red (*P. scapulatus*), and spectacled (*P. conspicillatus*) fruit bats (61,62; P.L. Young et al., unpub. results). Indeed the virus was antigenically and genetically indistinguishable from the earlier horse and human isolates. Thus, it is now clearly established that Hendra virus is a fruit bat virus and is widely distributed throughout the range of pteropid bats in Australia, with serologic evidence of infection in an average of 42% of wild-caught bats, the number of seropositive animals varying with species (53% of 229 *P. alecto,* 47% of 195 *P. poliocephalus*, 12% of 115 *P. scapulatus*, and 41% of 99 *P. conspicillatus*) and age, but not with geographic distribution (H. Field, unpub. results). Serologic evidence of infection of fruit bats has also been reported from Papua New Guinea. Two species of antibody-positive bats (*Dobsonia moluccense*, *P. neohibernicus)* were identified from Madang on the north coast of Papua New Guinea (K. Halpin, H. Field, J.S. Mackenzie, M. Bockarie, P.L. Young, P.W. Selleck, unpub. results), and bats of four more species (*D. andersoni, P. capistratus, P. hypomelanus*, and *P. admiralitatum*) were identified in Port Moresby and New Britain (H. Field, S. Hamilton, L. Hall, F. Bornacosso, K. Halpin, P.L. Young, unpub. results).

Morphologic features (63) and preliminary sequencing data of the M and F genes (64) suggested that Hendra virus was a member of the *Paramyxoviridae*, although it had unusual surface projections of two distinct lengths, 15 nm and 18 nm (63). The entire genome of the virus has now been sequenced (65;66; L.F. Wang, B.T.

Eaton and colleagues, unpub. results) and has revealed a gene order and P gene organization characteristic of members of the *Paramyxovirus* and *Morbillivirus* genera (65). Comparison of its deduced amino acid sequences with those of other family members confirm that Hendra virus is a member of the subfamily *Paramyxovirinae,* more closely related to members of the *Paramyxovirus* and *Morbillivirus* genera than the *Rubulavirus* genus. Overall, homology with other members of the subfamily is lower than that observed within an individual genus (L.F. Wang, B.T. Eaton, unpub. results). Hendra virus has several distinguishing features, including a genome that is 15% larger than that of other members, with each of the six transcription units containing a very long 3' untranslated region (L.F. Wang, B.T. Eaton, unpub. results). The P/V/C gene has a fourth open reading frame located between those of the C and V proteins and potentially encoding a small basic protein similar to those of some members of the *Rhabdoviridae* and *Filoviridae*; its long 3' untranslated region is a common feature of the *Filoviridae* (65). The sequence of the N gene has also recently been described (66), and like the P/V/C gene, has a 3' untranslated region approximately tenfold longer than other members of the *Paramyxovirinae*. Although the deduced amino acid sequence of the N protein was slightly more homologous to members of the *Morbillivirus* genus than to those of other *Paramyxovirinae* genera, the level of identity was much lower than that observed within the *Morbillivirus* genus.

Three other findings differentiate Hendra from most other members of the *Paramyxoviridae*: the wide host range (59), the cleavage site of the F protein, and the orientation of the cell surface from which virus is released (B.T. Eaton, W. Michalski, and M. Williamson, unpub. results). An accumulating body of evidence—size of genome, comparative sequence analyses, coding capacity for a small basic protein in the P gene, morphologic features, host range, and various biologic properties, together with the wildlife host of the virus—suggests that the virus had been misnamed—it was neither an equine virus nor a morbillivirus—although the name was relevant when the virus was first isolated. It has therefore been suggested that the virus be renamed Hendra and be classified in a new genus within the *Paramyxovirinae* (59,65,66). A number of aspects of the ecology of the virus remain to be determined. For instance, despite the obvious ubiquity of the virus in the fruit bat population and the extremely close relationship between bat caregivers and bats, there is no evidence of seroconversions among caregivers, despite their close contact with up to 1,000 bats per year (50). Specimens of persons who have died of either pneumonia or encephalitis of unknown etiology were virus-negative (C. Allan, J.S. Mackenzie, L.A. Selvey, unpub. results). Furthermore, all human infections appear to have been transmitted by horses. Thus, the virus appears to have low transmissibility to humans; it also appears to be linked with pregnancy: the index case of the Brisbane outbreak was a pregnant mare, a pregnant mare was involved in the Mackay incident, both incidents occurred during the birthing season of flying foxes, and virus was first recovered from uterine fluid of a pregnant animal (3). Thus, a number of questions remain about the ecologic, biologic, and pathologic characteristics of Hendra virus: 1) the infectivity and virulence of the virus and why it seems extremely difficult to transmit naturally between and within some susceptible host species, 2) classification of the virus, 3) role of pregnancy to transmission of the virus, 4) role of prior infection in horses in human infection, 5) method of transmission between fruit bats and from fruit bats to horses, 6) tropism of the virus, and 7) potential for producing a latent infection in humans.

### Australian Bat Lyssavirus

Australia had been considered free of rabies and rabieslike viruses until 1996 when a new lyssavirus, closely related to classic rabies virus, was first identified in a fixed brain specimen from a young black flying fox (*P. alecto*), with unusual neurologic symptoms. Since this original isolation, a further 42 isolates have been obtained from all four species of fruit bat, with most isolates from black and little red (*P. scapulatus*) flying foxes, and four isolates from an insectivorous bat (*Microchiroptera*), the yellow-bellied sheathtail bat (*Saccolaimus flavicentris*) (P. Daniels, R. Lunt, unpub. results). The isolates were from as far apart as Melbourne and Darwin, but most were from Queensland. Antibodies to rabies virus (REFIT assay) have been detected in 2.6% of 345 bat nonrandom samples submitted to the Australian Animal Health Laboratory (P. Daniels, R. Lunt, unpub. results). Antibody has been detected in both infected and apparently healthy bats, but whether this reflects the ability of bats to recover from infection or become latently infected with the virus is not known.

Analysis using nucleocapsid-specific monoclonal antibodies showed a strong relationship between this new lyssavirus and serotype 1 rabies virus (67). Indeed, rabies vaccine may elicit a protective immune response in humans, indicating the antigenic similarity of Australian bat lyssavirus and classic rabies virus (P.K. Murray, pers. comm. to the Lyssavirus Expert Committee [68]). Phylogenetic studies of the N protein sequences indicated that the Australian virus was genetically distinct from classic rabies (genotype 1) and was, therefore, a previously unrecognized member of the *Lyssavirus* genus and represented a new genotype, genotype 7 (67).

Two human infections have been attributed to Australian bat lyssavirus. One fatal rabieslike infection was in a female bat caregiver from Rockhampton, Queensland (69,70). An isolate of Australian bat lyssavirus obtained post-mortem was antigenically and genetically similar to the virus from the insectivorous yellow-bellied sheathtail bat (A.R. Gould, R. Lunt, P. Daniels, unpub. results). A second death has recently been reported in Queensland (J. Hann, J. Faoagali, G. Smith, I Serafin, J. Northill, unpub. obs.). The infection was in a 27-year-old woman from Mackay, who died 2 years after a bat bite (by a large flying fox). Polymerase chain reaction (PCR) testing of RNA extracted from saliva and nuchal biopsy proved vital to the antemortem diagnosis. Immunofluorescence staining of postmortem samples confirmed the diagnosis. Preliminary sequencing of the amplicon has indicated that the virus is very similar to other lyssaviruses isolated from flying foxes but clearly distinct from a virus isolate from a yellow-bellied sheathtail bat (I. Serafin, G. Smith, J. Hanna, B. Harrower, J. Northill, A. Westcott, unpub. obs.). More extensive sequencing of the human and bat isolates is under way. Measures to prevent further human infection have been implemented (68,71).

As with Hendra virus, a number of questions remain about the ecology and biology of Australian bat lyssavirus. The finding of well, antibody-positive bats, which suggests that bats can either recover from infection or that they can be silently infected, needs to be investigated, particularly with respect to infectivity and possible transmissibility. More information is needed on the geographic and host range of the virus ecology within bat communities and risk for transmission to terrestrial animals.

These novel zoonotic viruses appear to have frugivorous bats as their natural vertebrate hosts. While little is known of the viral fauna of fruit bats (or indeed of most wildlife species) in Australia, the occurrence of these three zoonotic viruses from bats over 3 years suggests that further prospective studies of diseases of wildlife are warranted. Indeed, two paramyxoviruslike viruses unrelated to any other known paramyxoviruses (K. Halpin, P.L. Young, unpub. results) have recently been isolated from flying foxes.

### Conclusions

The vector-borne and zoonotic diseases in this editorial encompass three patterns of emergence: known diseases increasing in incidence or geographic range (e.g., dengue and JE virus, respectively); new infectious agents as etiologic agents of known diseases (e.g., Australian bat lyssavirus as a cause of a rabieslike illness); and new infectious agents causing previously unrecognized diseases (e.g., Hendra virus). All three patterns demonstrate the need (and international responsibility) for ongoing surveillance and monitoring. In Australia, surveillance is the legislative responsibility of the individual states and territories. A Communicable Diseases Network Australia-New Zealand was established in 1989 to improve the control of communicable diseases in Australia by coordinating national surveillance activities and responses to outbreaks and by training public health staff. In 1996, Australia developed a National Communicable Diseases Surveillance Strategy to provide a national framework to monitor infectious diseases and plan and prioritize interventions. Components of the strategy include improvements to the national surveillance infrastructure, better monitoring of diseases and surveillance data, and better response to outbreaks. The strategy is being implemented and may provide the mechanism for a national response to new and reemerging diseases.

### Acknowledgments

I thank my many colleagues—Bryan Eaton, Lin-Fa Wang, Peter Daniels, Ross Lunt, Jeffrey Hanna, Scott Ritchie, Peter Young, Kim Halpin, Greg Smith, Debbie Phillips, Cheryl Johansen, Hume Field, Steve Flew, and John Oakley—whose unpublished work I have been permitted to quote.

### References

1. Beaman MH. Emerging infections in Australia. Ann Acad Med Singapore 1997;26:609-15.
2. Longbottom H. Emerging infectious diseases. Commun Dis Intell 1997;21:89-93.
3. Mackenzie JS, Bolton W, Cunningham AL, Frazer IH, Gowans EJ, Grohmann GS, et al. Emerging viral diseases of humans: an Australian and New Zealand perspective. In: Asche V, editor. Recent advances in microbiology. Vol. 5. Melbourne: Australian Society for Microbiology Inc.; 1997. p. 13-130.
4. Mackenzie JS. Emerging viral diseases: some comments from a regional perspective. P N G Med J. In press 1998.
5. Della-Porta AJ. Emerging and re-emerging diseases of animals in Australia. In: Asche V, editor. Recent advances in microbiology. Vol. 5. Melbourne: Australian Society for Microbiology Inc.; 1977. p. 131-201.
6. Desmarchelier PM. Foodborne disease: emerging problems and solutions. Med J Aust 1996;165:668-71.
7. Crerar SK, Dalton CB, Longbottom HM, Kraa E. Foodborne disease: current trends and future surveillance needs in Australia. Med J Aust 1996;165:672-5.
8. Collignon PJ. Antibiotic resistance: is it leading to the re-emergence of many infections of the past? In: Asche V, editor. Recent advances in microbiology. Vol. 5. Melbourne: Australian Society for Microbiology Inc.; 1997. p. 203-56.
9. Collignon PJ, Bell JM. Drug-resistant *Streptococcus pneumoniae*: the beginning of the end for many antibiotics? Australian Group on Antimicrobial Resistance. Med J Aust 1996;164:64-7.
10. Heath CH, Blackmore TK, Gordon DL. Emerging resistance in *Enterococcus* spp. Med J Aust 1996;164:116-20.
11. Maguire GP, Arthur AD, Boustead PJ, Dwyer B, Currie BJ. Emerging epidemic of community-acquired methicillin-resistant *Staphylococcus aureus* infection in the Northern Territory. Med J Aust 1996;164:721-3.
12. Gratten M, Nimmo G, Carlisle J, Schooneveldt J, Seneviratne E, Kelly R, et al. Emergence of further serotypes of multiple drug-resistant *Streptococcus pneumoniae* in Queensland. Commun Dis Intell 1997;21:133-6.
13. Dawson D. Tuberculosis in Australia: bacteriologically confirmed cases and drug resistance, 1996. Report of the Australian Mycobacterium Reference Laboratory Network. Commun Dis Intell 1998;22:183-7.
14. Givney R, Vickery A, Holliday A, Pegler M, Benn R. Evolution of an endemic methicillin-resistant *Staphylococcus aureus* population in an Australian hospital from 1967 to 1996. J Clin Microbiol 1998;36:552-6.
15. Maguire GP, Arthur AD, Boustead PJ, Dwyer B, Currie BJ. Clinical experience and outcomes of community-acquired and nosocomial methicillin-resistant *Staphylococcus aureus* in a northern Australian hospital. J Hosp Infect 1998;38:273-81.
16. Grimwood K, Collignon PJ, Currie BJ, Ferson MJ, Gilbert GL, Hogg GG, et al. Antibiotic management of pneumococcal infections in an era of increased resistance. J Paediatr Child Health 1997;33:287-95.
17. Patel MS, Collignon PJ, Watson CR, Condon RJ, Doherty RR, Merianos A, et al. New guidelines for management and prevention of meningococcal disease in Australia. Meningococcal Disease Working Party of the National Health and Medical Research Council. Med J Aust 1997;166:598-601.
18. Cameron S, Walker C, Beers M, Rose N, Anear E. Enterohaemorrhagic *Escherichia coli* outbreak in South Australia associated with the consumption of mettwurst. Commun Dis Intell 1995;19:70-1.
19. Ng S, Rouch G, Dedman R, Harries B, Boyden A, McLennan L, et al. Human salmonellosis in peanut butter. Commun Dis Intell 1996;20:326-7.
20. Hook D, Jalaludin B, Fitzsimmons G. *Clostridium perfringens* food-borne outbreak: an epidemiological investigation. Aust N Z J Public Health 1996;20:119-22.
21. Stafford R, Strain D, Heymer M, Smith C, Trent M, Beard J. An outbreak of Norwalk virus gastroenteritis following consumption of oysters. Commun Dis Intell 1997;21:317-20.
22. Salmonellosis outbreak. Commun Infect Dis 1998;22:155.
23. Lemmon JM, McAnulty JM, Bawden-Smith J. Outbreak of cryptosporidiosis linked to an indoor swimming pool. Med J Aust 1996;165:613-6.
24. Cryptosporidiosis outbreak. Commun Dis Intell 1998;22:22.
25. Parasites in water. Commun Dis Intell 1998;22:190.
26. Hyland CA, Mison L, Solomon N, Cockerill J, Wang L, Hunt J, et al. Exposure to GBV-C/HGV in selected Australian adult and children populations. Transfusion. In press 1998.
27. Mackenzie JS, Broom AK, Hall RA, Johansen CA, Lindsay MD, Phillips DA, et al. Arboviruses in the Australian region, 1990 to 1998. Commun Dis Intell 1998;22:93-100.
28. Mackenzie JS, Lindsay MD, Coelen RJ, Broom AK, Hall RA, Smith DW. Arboviruses causing human disease in the Australasian zoogeographic region. Arch Virol 1994;136:447-67.
29. Lindsay MDA, Johansen CA, Broom AK, Smith DW, Mackenzie JS. Emergence of Barmah Forest virus in Western Australia. Emerg Infect Dis 1995;1:22-6.
30. Lindsay MD, Johansen CA, Smith DW, Wallace MJ, Mackenzie JS. An outbreak of Barmah Forest virus disease in the south-west of Western Australia. Med J Aust 1995;162:291-4.
31. Hanna J, Ritchie S, Phillips DA, Shield J, Bailey MC, Mackenzie JS, et al. An outbreak of Japanese encephalitis in the Torres Strait, Australia, 1995. Med J Aust 1996;256-60.
32. Ritchie SA, Phillips D, Broom A, Mackenzie J, Poidinger P, Van Den Hurk A. Isolation of Japanese encephalitis virus from *Culex annulirostris* mosquitoes in Australia. Am J Trop Med Hyg 1997;56:80-4.
33. Mackenzie JS, Poidinger M, Phillips D, Johansen C, Hall RA, Hanna J, et al. Emergence of Japanese encephalitis virus in the Australasian region. In: Saluzzo JF, Dodet B, editors. Factors in the emergence of arboviral diseases. Paris: Elsevier; 1997. p. 191-201.
34. Poidinger M, Hall RA, Mackenzie JS. Molecular characterisation of the Japanese encephalitis serocomplex of the *Flavivirus* genus. Virology 1996;218:417-21.
35. Hanna J, Barnett D, Ewaid D. Vaccination against Japanese encephalitis in the Torres Strait. Commun Dis Intell 1996;20:188-90.
36. Shield J, Hanna J, Phillips D. Reappearance of the Japanese encephalitis virus in the Torres Strait, 1996. Commun Dis Intell 1996;20:191.

37. Johansen C, Ritchie S, Van Den Hurk A, Bockarie M, Hanna J, Phillips D, et al. The search for Japanese encephalitis virus in the Western province of Papua New Guinea. In: Kay BH, Brown MD, Aaskov JG, editors. Arbovirus research in Australia. Vol. 7. Brisbane: Queensland Institute of Medical Research; 1997. p. 131-6.
38. Japanese encephalitis on the Australian mainland. Commun Dis Intell 1998;22:60.
39. Mackenzie JS. Japanese encephalitis: an emerging disease in the Australian region, and its potential risk to Australia. In: Kay BH, Brown MD, Aaskov JG, editors. Arbovirus research in Australia. Vol. 7. Brisbane: Queensland Institute of Medical Research; 1997. p. 166-70.
40. Mackenzie JS, LaBrooy JT, Hueston L, Cunningham AL. Dengue in Australia [editorial]. J Med Microbiol 1996;45:159-61.
41. Sinclair DP. The distribution of *Aedes aegypti* in Queensland, 1990 to 30 June 1992. Commun Dis Intell 1992;16:400-3.
42. Phillips D, Pearce M, Weimers M, Blumke G. Dengue 2 infection in northern Queensland. Commun Dis Intell 1992;16:192-3.
43. Row D, Pearce M, Hapgood G, Sheridan J. Dengue and dengue haemorrhagic fever in Charters Towers, Queensland. Commun Dis Intell 1993;17:182-3.
44. Griffiths M, Ritchie S, Terry D, Norton R, Phillips D. An outbreak of dengue 2 in the Torres Strait. Commun Dis Intell 1997;21:33.
45. Hanna JN, Ritchie SA, Merritt AD, Van den Hurk AF, Phillips DA, Serafin IL, et al. Two contiguous outbreaks of dengue type 2 in north Queensland. Med J Aust 1998;168:221-5.
46. Dengue 3 in Cairns: the story so far. Commun Dis Intell 1998;22:109-10.
47. Ritchie S, Hanna J, Van den Hurk A, Harley D, Lawrence R, Phillips D. Importation and subsequent local transmission of dengue 2 in Cairns. Commun Dis Intell 1995;19:366-70.
48. Hanna J, Ritchie S, Tiley S, Phillips D. Dengue imported from Papua New Guinea. Commun Dis Intell 1995;19:447.
49. Foley P, Hemslaey C, Muller K, Maroske G, Ritchie S. Importation of *Aedes albopictus* in Townsville, Queensland. Commun Dis Intell 1998;22:3-4.
50. Selvey L, Taylor R, Arklay A, Gerrard J. Screening of bat carers for antibodies to equine morbillivirus. Commun Dis Intell 1996;20:477-8.
51. Philbey AW, Kirkland PD, Ross AD, Davies RJ, Gleeson AB, Love RJ, et al. An apparently new virus (family Paramyxoviridae) infectious for pigs, humans, and fruit bats. Emerg Infect Dis 1998;4:269-71.
52. Chant K, Chan R, Smith M, Dwyer DE, Kirkland P, the NSW Expert Group. Probable human infection with a newly described virus in the family Paramyxoviridae. Emerg Infect Dis 1998;4:273-5.
53. Murray K, Rogers R, Selvey L, Selleck P, Hyatt A, Gould A, et al. A novel morbillivirus pneumonia of horses and its transmission to humans. Emerg Infect Dis 1995;1:31-3.
54. Murray K, Selleck P, Hooper P, Hyatt A, Gould A, Gleeson L, et al. A morbillivirus that caused fatal disease of horses and humans. Science 1995;268:94-6.
55. Selvey LA, Wells RM, McCormack JG, Ansford AJ, Murray K, Rogers RJ, et al. Infection of humans and horses by a newly described morbillivirus. Med J Aust 1995;162:642-5.
56. Rogers RL, Douglas IC, Baldock FC, Glanville RJ, Seppanen KT, Gleeson LJ, et al. Investigation of a second focus of equine morbillivirus infection in coastal Queensland. Aust Vet J 1996;74:243-4.
57. O'Sullivan JD, Allworth AM, Paterson DL, Snow TM, Boots R, Gleeson LJ, et al. Fatal encephalitis due to a novel paramyxovirus transmitted from horses. Lancet 1997;349:93-5.
58. Hooper PT, Gould AR, Russell GM, Kattenbelt JA, Mitchell G. The retrospective diagnosis of a second outbreak of equine morbillivirus infection. Aust Vet J 1996;74:244-5.
59. Murray K, Eaton B, Hooper P, Wang L, Williamson M, Young P. Flying foxes, horses, and humans: a zoonosis caused by a new member of the *Paramyxoviridae*. In: Scheld WM, Armstrong D, Hughes JM, editors. Emerging infections 1. Washington: American Society for Microbiology Press; 1998. p. 43-58.
60. Ward MP, Black PF, Childs AJ, Baldock FC, Webster WR, Rodwell BJ, et al. Negative findings from serological studies of equine morbillivirus in the Queensland horse population. Aust Vet J 1996;74:241-3.
61. Young PL, Halpin K, Selleck PW, Field H, Gravel JL, Kelly MA, et al. Serologic evidence for the presence in pteropus bats of a paramyxovirus related to equine morbillivirus. Emerg Infect Dis 1996;2:239-40.
62. Young P, Halpin K, Field H, Mackenzie J. Finding the wildlife reservoir of equine morbillivirus. In: Asche V, editor. Recent advances in microbiology. Vol. 5. Melbourne: Australian Society for Microbiology Inc.; 1997. p. 1-12.
63. Hyatt AD, Selleck PW. Ultrastructure of equine morbillivirus. Virus Res 1996;43:1-15.
64. Gould AR. Comparison of the deduced matrix and fusion protein sequences of equine morbillivirus with cognate genes of the Paramyxoviridae. Virus Res 1996;43:17-31.
65. Wang LF, Michalski WP, Yu M, Pritchard LI, Crameri G, Shiell B, et al. A novel P/V/C gene in a new member of the *Paramyxoviridae* family, which causes lethal infection in humans, horses, and other animals. J Virol 1998;72:1482-90.
66. Yu M, Hansson E, Shiell B, Michalski W, Eaton BT, Wang LF. Sequence analysis of the Hendra virus nucleoprotein gene: comparison with other members of the subfamily Paramyxoviridae. J Gen Virol 1998;79:1775-80.
67. Gould AR, Hyatt AD, Lunt R, Kattenbelt JA, Hengstberger S, Blacksell SD. Characterisation of a novel lyssavirus isolated from Pteropid bats in Australia. Virus Res 1998;54:165-87.
68. Lyssavirus Expert Group. Prevention of human lyssavirus infection. Commun Dis Intell 1996;20:505-7.
69. Hooper PT, Lunt RA, Gould AR, Samaratunga H, Hyatt AD, Gleeson LJ, et al. A new lyssavirus–the first endemic rabies-related virus recognized in Australia. Bulletin de l'Institut Pasteur 1997;95:209-18.
70. Allworth A, Murray K, Morgan J. A case of encephalitis due to a lyssavirus recently identified in fruit bats. Commun Dis Intell 1996;20:504.
71. Lyssavirus Expert Group. Update on bat lyssavirus. Commun Dis Intell 1996;20:535.
